# Determining Thermal Conductivity of Small Molecule Amorphous Drugs with Modulated Differential Scanning Calorimetry and Vacuum Molding Sample Preparation

**DOI:** 10.3390/pharmaceutics11120670

**Published:** 2019-12-10

**Authors:** Maximilian Karl, Jukka Rantanen, Thomas Rades

**Affiliations:** 1 Department of Pharmacy, University of Copenhagen, Universitetsparken 2, 2100 Copenhagen, Denmark; maximilian.karl@sund.ku.dk (M.K.); jukka.rantanen@sund.ku.dk (J.R.); 2 Danish National Research Foundation and Villum Fondens Center for Intelligent Drug Delivery and Sensing Using Microcontainers and Nanomechanics (IDUN), Kgs. Lyngby, 2800 Copenhagen, Denmark

**Keywords:** thermal conductivity, amorphous, glass, indomethacin, celecoxib, DSC, small molecule

## Abstract

Thermal conductivity is a material specific property, which influences many aspects of pharmaceutical development, such as processing, modelling, analysis, and the development of novel formulation approaches. We have presented a method to measure thermal conductivity of small molecule organic glasses, based on a vacuum molding sample preparation technique combined with modulated differential scanning calorimetry. The method is applied to the two amorphous model compounds indomethacin and celecoxib. The measured values of below 0.2 W/m °C indicate very low thermal conductivity of the amorphous compounds, within the range of organic liquids and low conducting polymers.

## 1. Introduction

The ability of a specific sample to conduct heat, measured as the material’s thermal conductivity ĸ, is an important parameter for many applications and theoretical considerations. Whilst the determination of thermal conductivity of large molecule samples, such as polymers, is a long-established procedure in many fields of material sciences [1], there is a considerable literature gap for small molecule organic glasses, especially active pharmaceutical ingredients (API).

However, converting an initially crystalline API to its amorphous form is a promising strategy to counteract the drug development challenge of low aqueous solubility, especially if oral drug delivery is preferred [2,3]. Determining thermal conductivity is thus becoming more interesting and a necessity in several methods related to the research and development of amorphous API. Thermal properties are particularly important in drug processing. Milling, for example, can induce a mechanically activated disordering of the crystalline API and thermal conductivity strongly influences the temperature increase needed for complete amorphisation [4]. Furthermore, developing novel formulation approaches such as in situ amorphisation—For example, the conversion of a drug to its amorphous form prior to administration to the patient with the help of microwave heating [5]—Would benefit from having established thermal conductivity values.

Moreover, thermal conductivity is an important input parameter in many solid-state models, including models for process flow, e.g., in hot melt extrusion [6,7], or models to help understand the material responses to recently developed analytical techniques [8].

Thermal conductivity and diffusivity meters, which often utilize the flash- or guarded heat flow method, are rarely part of general physicochemical characterization equipment, especially in pharmaceutical laboratories. Small-scale sample availability, sample geometry or the accessible temperature range can further limit the use of these instruments. Therefore, it is the aim of this communication to offer a practical guide to a more readily available alternative for heat conductivity measurements of small molecule API glasses. The method is based on combining vacuum molding sample preparation with regular modulated differential scanning calorimetry (mDSC), utilizing a previously reported method [9]. Sample preparation tools using vacuum compression molding are increasingly becoming part of the standard analytical equipment park within pharmaceutical research and development [10,11,12] and DSC is a regular part of sample characterization, in particular for amorphous compounds. Several methods and a few application notes, published by thermal equipment manufacturers, using DSC to measure thermal conductivity are described in the literature [9,13,14,15,16,17].

However, all of these studies focus on well-known polymeric samples. Only one study, which employs a similar DSC method to this communication, targeted small molecule samples, such as active pharmaceutical ingredients. The aim of that study was not the preparation and measurement of small molecule organic glasses but rather a thermal conductivity estimation of a compacted powder specimen with an added correction for sample porosity [18].

Consequently, in this study, we demonstrated the feasibility of vacuum molding sample preparation and DSC for thermal conductivity measurements of the two amorphous model compounds indomethacin (IND) and celecoxib (CCX).

The applied measurement technique was based on the possibility of mDSC to directly measure heat capacity. In a regular mDSC run, accurate heat capacity results are obtained when the experimental conditions facilitate maximum temperature uniformity across the sample specimen. Therefore, standard mDSC runs are performed with long modulation periods and a thin specimen encapsulated in a pan of high thermal conductivity (see also “thin sample” measurement below). If these conditions are not met, the measured heat capacity decreases, mainly because the thermal conductivity of the sample is preventing temperature uniformity [9]. To maximize this effect, a thick sample can be measured without encapsulation (see also “thick sample” measurement below) and the sample’s thermal conductivity can be estimated from the difference of the two obtained heat capacity values.

## 2. Materials and Methods

### 2.1. Materials

IND was purchased from Tokyo Chemical Industry Co., Ltd. (Tokyo, Japan), CCX was obtained from Fagron, Inc. (St. Paul, MN, USA), polystyrene (PS, Mw ~ 192 k) and polymethylmethacrylate (PMMA, Mw ~ 120 k) were purchased from Sigma-Aldrich (St. Louis, MO, USA).

### 2.2. Sample Preparation

**Figure 1:**
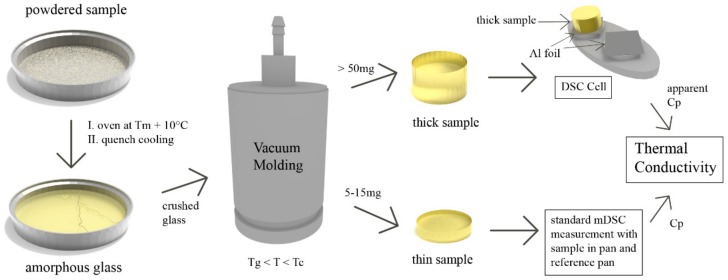
Sample preparation process and measurement schematic for the small molecule amorphous specimen.

The sample preparation process (and measurement) for the small molecule samples is presented in Figure 1. The sample was first converted from its crystalline state to an amorphous form by standard quench cooling. This comprised of covering an aluminium pan with sample powder, heating it 10 °C above its respective melting point in an oven for 5 min and quickly cooling the molten sample afterwards by transferring the pan onto a cold surface such as a metal bench. This initial amorphisation was necessary because many small molecule samples possess a very low melt viscosity (0.05 and 0.07 Pas for CCX and IND, respectively [19]) for vacuum molding, leading to non-uniform samples. By pre-quenching (i.e., amorphising) the sample, vacuum molding can be performed at temperatures above the glass transition (Tg) but below the crystallization temperature (Tc). The viscosity at temperatures in the supercooled melt of most APIs is sufficiently high to produce uniform samples [19]. Vacuum molding to obtain cylindrical samples was performed with the MeltPrep^®^ system (MeltPrep^®^ GmbH, Graz, Austria). In this study, the crushed glass was transferred into the molding tool (5 mm diameter disc tool) and kept at 10–30 °C above the glass transition for 12 min (termed: “thick sample”) or 10 min (termed “thin sample”). Glass transition temperature values were obtained beforehand by a single measurement on a standard DSC (see supporting information for DSC thermograms, Figure S1). To obtain the “thick samples”, 50 to 70 mg of crushed glass was used, while 5–15 mg was used for the “thin samples”. Cooling was performed on the implemented cooling device without active water cooling.

Samples were confirmed to be amorphous by X-ray powder diffraction (XRPD). The polymeric samples (PS and PMMA) were directly filled into the molding tool without any sample pretreatment and heated 30 to 40 °C above their respective glass transition temperature for 10 min.

### 2.3. Modulated Differential Scanning Calorimetry

Measurements on a Discovery DSC (TA Instruments, New Castle, DE, USA) were performed in triplicate and the overall DSC method was adapted from the standard test method E1952-17 [20], which was related to references [9,13]. The reader is referred to the standard test method for an overview including performance criteria alongside a study on precision and bias. The DSC in the modulated mode was calibrated beforehand for heat capacity measurements with a TA Instruments sapphire calibration disc in temperature-dependent calibration [21]. The method is summarized in the following steps (see also Figure 1):

1. The heat capacity of the “thin sample” (Cp,s) was measured in a standard run with the sample inside a DSC pan and an empty pan on the reference side.
2. The “thick sample” was weighed and its length and diameter were measured with a caliper. The apparent heat capacity of the “thick sample” (Cp,app) was measured by placing the sample on the sample side of the DSC cell. A piece of aluminium foil with a small amount of silicone oil (wetted cotton swab to apply) was placed in between the sample and cell. A similar foil was placed on the reference side. The mass of the “thick sample” was entered in the DSC software as the sample mass.
3. The thermal conductivity was calculated with the help of Cp,s, Cp,app, as well as mass, length and diameter of the “thick sample”. The equations that were used have been supplied in Section 2.4.

The DSC method (for the estimation of both Cp,s and Cp,app) consisted of an equilibration step at the measurement temperature followed by a 5 min isothermal step. Afterwards, data was collected over another 5-min isothermal interval. A modulation amplitude of 0.5 °C and a period of 80 s were used for all measurements in this study. The measurement procedure was first performed with a sample of known thermal conductivity (a polystyrene reference from the thermal conductivity kit supplied by TA Instruments, P/N 915064.901) to obtain the calibration factor D. Every sample measurement was subsequently corrected by this factor. To test the preparation method and measurement performance, the two polymeric samples PMMA and PS (obtained as granules) were vacuum molded and measured as described above. The PS sample was measured over a broader temperature range to test and compare the method and its accuracy to E1952-17 and validate the calibration factor at measurement temperatures. A single point measurement of PMMA (a compound with well reported literature values for thermal conductivity [22,23]) was used to further qualify the method performance. Temperatures were kept well below the respective Tg temperatures to avoid the contamination of the DSC cell from the “thick samples” due to liquefaction of the small molecule API samples.

### 2.4. Equations Used to Calculate the Sample’s Thermal Conductivity

I. The measured thermal conductivity Km (W/m °C) was calculated by:

(1) $$K_{m}=\;\frac{8\;l\;{\mathrm{Cp}}_{\mathrm{app}}^{2}}{{\mathrm{Cp}}_{,s}\;m\;d^{2}\;P}$$

l: sample length (mm); ${\mathrm{Cp}}_{,s}$: specific heat capacity $\left(\frac{J}{g{}^{\circ}C}\right)$; ${\mathrm{Cp}}_{\mathrm{app}}$: “thick sample” apparent heat capacity $\left(\frac{\mathrm{mJ}}{{}^{\circ}C}\right)$; m: “big sample “ mass (mg); d: big sample diameter (mm); P: period (s).

II. The sample’s thermal conductivity K_S_ (W/m °C) was calculated by:

(2) $$K_{s}=\;\frac{K_{m}-2D+{\left(K_{m}^{2}-4DK_{m}\right)}^{\frac{1}{2}}}{2}$$

with *D* (determined with K_m_ of a sample of known thermal conductivity by Equation (1)):

(3) $$D\;={\left(K_{m}K_{r}\right)}^{\frac{1}{2}}-K_{r}$$

$K_{r}:\mathrm{reference}\;\mathrm{value}\;\left(\frac{W}{m{}^{\circ}C}\right).$

### 2.5. X-ray Powder Diffraction (XRPD)

The sample discs were crushed and grinded prior to the XRPD measurements. The measurements were performed on a PANalytical X’Pert PRO diffractometer (PW3040/60, Alemo, The Netherlands) equipped with a Cu Kα anode (current: 30 mA, voltage: 45 kV) in the range of 4−34° 2θ.

## 3. Results

**Figure 2:**
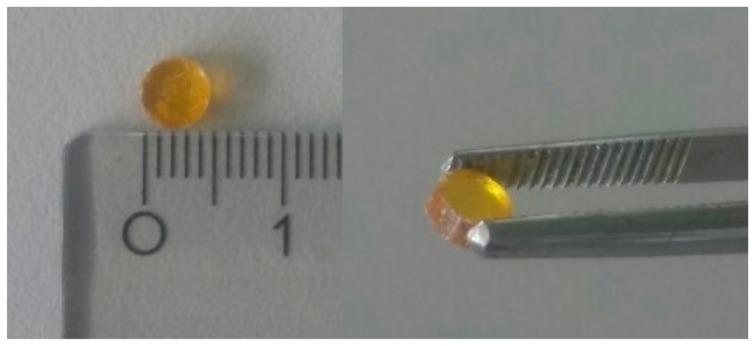
Amorphous indomethacin, specimen for Cp,app determination with a 5 mm diameter and 3.2 mm height.

After preparation, the small molecule API samples were fully X-ray amorphous. The reader is referred to the supporting information for example diffractograms with crystalline references (Figure S2). Samples produced by the vacuum molding process were without visible air voids and well defined in geometry (see Figure 2) and therefore allowed precise thermal conductivity measurements.

Table 1 lists the Tg values, molding temperatures and thermal conductivity values for all samples in this study. As can be seen by the PS and PMMA samples, the method produced thermal conductivity values, which were in agreement with literature, within the precision limits reported earlier [9].

**Figure 3:**
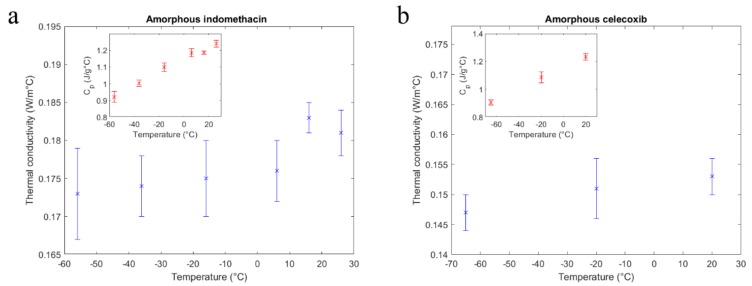
Thermal conductivity of indomethacin (**a**) and celecoxib (**b**). The insets show the specific heat capacity at the same measurement temperatures for the compounds, respectively.

The measured thermal conductivity alongside the measured heat capacity of the small molecule organic amorphous pharmaceuticals is further presented in Figure 3a,b.

As seen in the figure insets, the specific heat capacity of both compounds increased with temperature and where available, the absolute values were in agreement with literature [25]. In the measured temperature range, thermal conductivity values obtained for IND and CCX did not indicate a clear temperature dependence.

A small increase in thermal conductivity with temperature was visible for both compounds, similar to many low conducting glasses and polymers [26]. However, a clear interpretation of these minute changes of an already low conducting material was outside of the method’s precision limits.

## 4. Discussion

With values below 0.2 W/m °C, the thermal conductivities of the small molecule samples were comparable to other disordered materials like low conducting polymers, as well as common organic liquids [26,27]. After the literature review and to the best of the authors’ knowledge, there were no thermal conductivity reference values available for the two amorphous drugs tested in this study. A further discussion on the absolute values is therefore limited. The local maximum in thermal conductivity of IND at 16 °C was most likely due to a small drop in heat capacity of the measured small specimen at this temperature (see also Figure 3a, inset).

Since thermal conductivity can be an important parameter in pharmaceutical manufacturing but is rather difficult to measure without specific instrumentation, estimates are often used for models describing specific processes. For example, an approximation of 0.18 W/m °C is made in a hot-melt extrusion numerical simulation using a model-based melt viscosity [7]. This study investigated binary amorphous solid dispersions of small molecule organic drugs with vinylpyrrolidone-vinyl acetate copolymer. While not having performed measurements on the described solid dispersions, our study indicated that values between 0.15–0.2 W/m °C were indeed fitting estimates for these systems. Furthermore, since absolute thermal conductivity values of the small molecule API were comparable to amorphous polymers, at lower API concentrations, reasonable estimates might be obtained from the polymer’s thermal conductivity only.

With special DSC tools available, lab bench molding equipment can provide a more practical sample preparation approach than other methods, such as specimen cutting from quarter-inch extruded or molded rods [9]. While polymeric samples are easily formed without further pre-treatment, small molecule samples are more challenging due to the possible need for pre-quenching and because the obtained glasses can be very fragile.

## 5. Conclusions

In this study, we demonstrated a method of preparing and analysing small molecule organic glasses for thermal conductivity measurements with mDSC. Vacuum molding was used to obtain well-defined samples and with the help of a previously described mDSC method, we were able to obtain thermal conductivity values for the two amorphous model compounds indomethacin and celecoxib. The values fell within the range of lower conducting disordered materials. Our study highlights the feasibility of vacuum molding and mDSC in providing thermal conductivity values of small molecule drug glasses for practical and theoretical considerations. The described approach could also be extended to drug polymer binary glass solutions.

## Figures and Tables

**Table 1:** Glass transition temperature (Tg), molding temperature and thermal conductivity (K) values obtained in this study.

Samples	Tg_, DSC_ [°C]	Molding Temperature [°C]	Measurement Temperature [°C] ^a^	ĸ_DSC_ [W/m °C]	Literature Values for ĸ [W/m °C]
PMMA	104	142	+17	0.1985 ± 0.0008	0.190 ^b^
Polystyrene	105	135	−65 −25 +20	0.134 ± 0.008 0.144 ± 0.008 0.152 ± 0.09	0.1432 ^c^ 0.1474 ^c^ 0.1529 ^c^
Amorphous Celecoxib	57	68	−65 −20 +20	0.147 ± 0.003 0.151 ± 0.005 0.153 ± 0.003	N.A. N.A. N.A.
Amorphous Indomethacin	45	75	−56 −36 −16 +6 +16 +26	0.173 ± 0.006 0.174 ± 0.004 0.175 ± 0.005 0.176 ± 0.004 0.183 ± 0.002 0.181 ± 0.003	N.A. N.A. N.A. N.A. N.A. N.A.

N.A.: not available. ^a^ Temperature stability ±0.2 °C. ^b^ Reference [23]. ^c^ Values from reference [24], linear interpolation was necessary.

## Supplementary Materials

The following are available online at https://www.mdpi.com/1999-4923/11/12/670/s1, Figure S1: Single Differential Scanning Calorimetry (DSC) (none modulated) measurements to determine the respective sample glass transition temperatures, Figure S2: X-ray powder diffractograms of crystalline and amorphous (ground disc sample) celecoxib and indomethacin.
