# Supplementary material for: Determining Thermal Conductivity of Small Molecule Amorphous Drugs with Modulated Differential Scanning Calorimetry and Vacuum Molding Sample Preparation
Source: Pharmaceutics. 2019 Dec 10;11(12):670. doi: 10.3390/pharmaceutics11120670 (PMC6955963; doi:10.3390/pharmaceutics11120670)
Supplement: Supplementary file 1 [file pharmaceutics-11-00670-s001.pdf]

pharmaceutics

Supplementary Materials: Determining Thermal Conductivity of Small Molecule Amorphous Drugs with Modulated Differential Scanning Calorimetry and Vacuum Molding Sample Preparation

Maximilian Karl, Jukka Rantanen and Thomas Rades

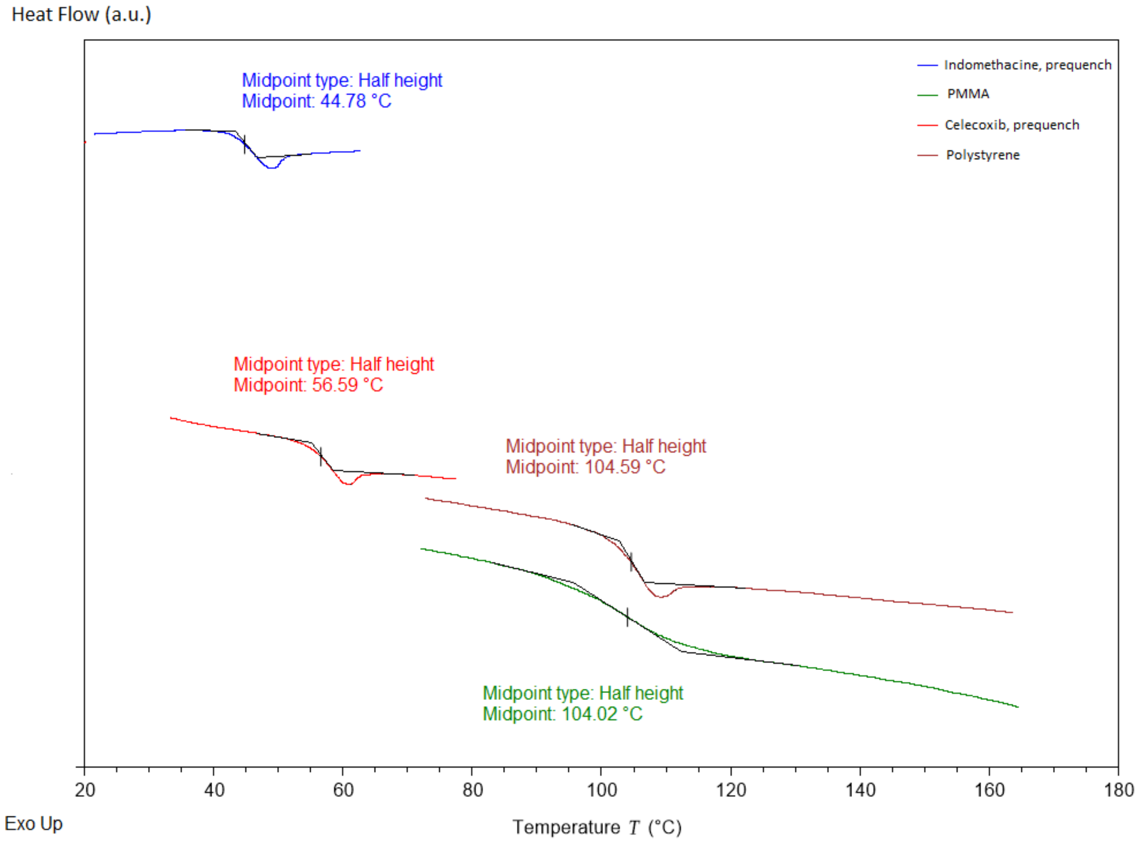

Figure S1. Single Differential Scanning Calorimetry (DSC) (none modulated) measurements to determine the respective sample glass transition temperatures.

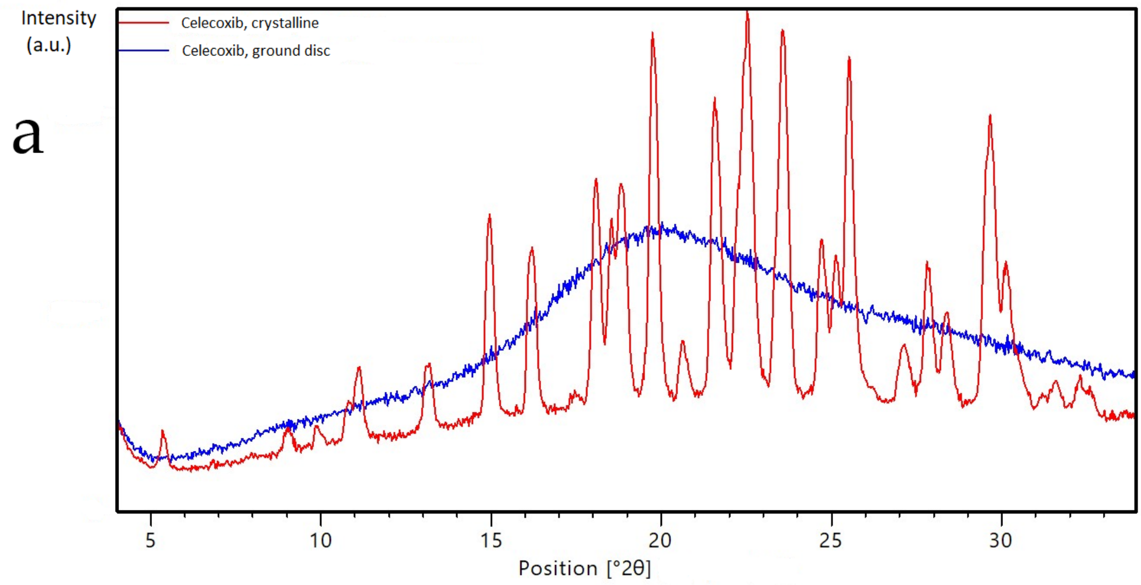

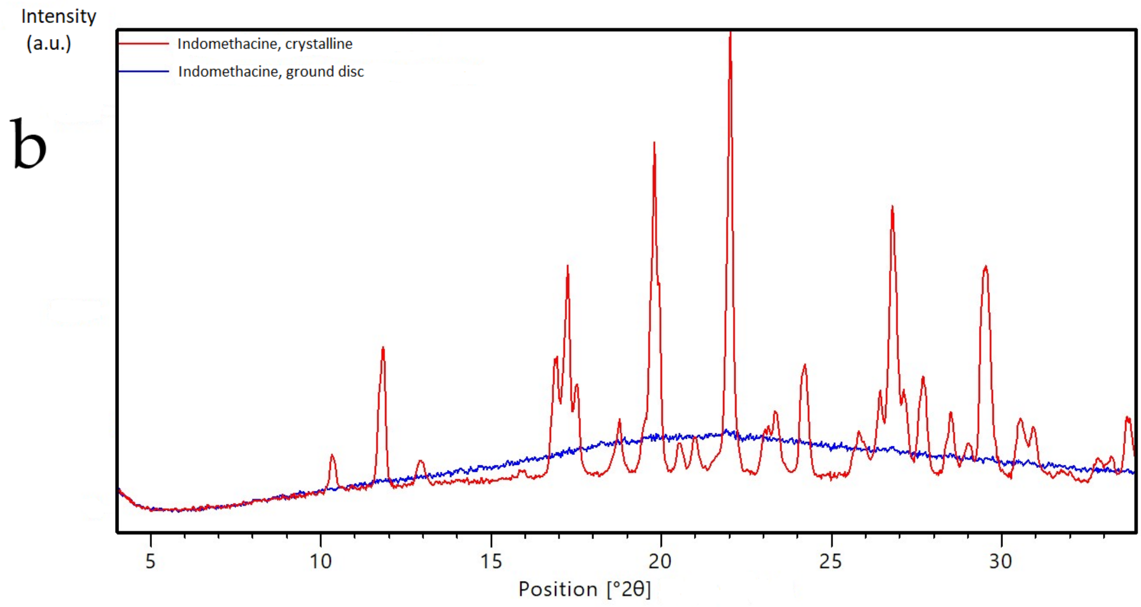

Figure S2. X-ray powder diffractograms of crystalline and amorphous (ground disc sample) celecoxib and indomethacin.
